# Effects of Cerebrolysin on Hippocampal Neuronal Death After Pilocarpine-Induced Seizure

**DOI:** 10.3389/fnins.2020.568813

**Published:** 2020-10-16

**Authors:** Dong Hyeon Kang, Bo Young Choi, Song Hee Lee, A Ra Kho, Jeong Hyun Jeong, Dae Ki Hong, Beom Seok Kang, Min Kyu Park, Hong Ki Song, Hui Chul Choi, Man-Sup Lim, Sang Won Suh

**Affiliations:** ^1^Department of Physiology, College of Medicine, Hallym University, Chuncheon, South Korea; ^2^Neurology, College of Medicine, Hallym University, Chuncheon, South Korea; ^3^Hallym Institute of Epilepsy Research, Chuncheon, South Korea; ^4^Department of Medical Education, College of Medicine, Hallym University, Chuncheon, South Korea

**Keywords:** epileptic seizure, pilocarpine, cerebrolysin, neurotropic, neuroprotective, neuropeptide, brain-derived neurotrophic factor

## Abstract

Epilepsy is one of the most common and severe brain diseases. The exact cause of epilepsy is unclear. Epilepsy often occurs following brain damage, such as traumatic brain injury (TBI) and ischemia. Cerebrolysin is a porcine brain peptide that is a unique neurotropic and neuroprotective agent. Cerebrolysin has been reported to increase neuroprotective effects after TBI, ischemia, and other CNS diseases. However, the effects of cerebrolysin on seizures are not known. Therefore, this study aimed to investigate the effects of neuropeptide cerebrolysin on neuronal death in the hippocampus after a seizure. To confirm the effects of cerebrolysin, we used a pilocarpine-induced seizure animal model. Cerebrolysin (2.5 ml/kg, i.p., once per day for 7 days) was immediately injected after a seizure induction. After 1 week, we obtained brain tissues and performed staining to histologically evaluate the potentially protective effects of cerebrolysin on seizure-induced neuronal death in the hippocampus. We found that cerebrolysin decreased hippocampal neuronal death after a seizure. In addition, an increase in brain-derived neurotrophic factor (BDNF) was confirmed through Western blot analysis to further support our hypothesis. Therefore, the present study suggests that the administration of cerebrolysin can be a useful therapeutic tool for preventing neuronal death after a seizure.

## Introduction

Epilepsy is one of the most common brain diseases, affecting about 70 million people worldwide (Thijs et al., 2019). Temporal lobe epilepsy (TLE) is the most common type of partial epilepsy, accounting for at least 20% of all patients with epilepsy (Babb, 1999). Although epilepsy has diverse subtypes and several co-occurring symptoms, the main causes of re-occurring epilepsy are the chronic downregulation of inhibitory neurotransmission or the overactivation of excitatory synaptic neurotransmission (Goldberg and Coulter, 2013). In addition, epilepsy often occurs after other brain diseases, such as traumatic brain injury and ischemia (Goldberg and Coulter, 2013). Thus, if the hippocampus is damaged by seizure, damage to the neuronal cells and changes in metabolic processes can result in the hippocampus failing to function normally (Holmes, 1991; Najm et al., 1998; Pitkanen and Sutula, 2002; Haut et al., 2004; Lukoyanov et al., 2004; Vingerhoets, 2006). Cognitive impairment and neuronal injury in epileptic patients remain important medical problems (Jeong et al., 2017; Lee S. H. et al., 2018). Various animal models have been developed to help identify therapeutic interventions to prevent these negative epileptic outcomes (Xie et al., 1995; Smolders et al., 2002; Remy et al., 2003). Thus, pilocarpine-induced seizure causes severe and extensive neuronal damage in the cerebral cortex and hippocampus (Turski et al., 1983; Leite et al., 1990; Lee M. et al., 2018).

The use of pilocarpine-induced seizures in rodents is an animal model that is commonly applied to the study of epilepsy (Levesque and Avoli, 2013; Lee et al., 2019; Wang et al., 2019). Pilocarpine acts on the M1 muscarinic receptor and serves as an agonist for the muscarinic acetylcholine receptor. When M1 is activated, phospholipase C is also activated, producing inositol triphosphate (IP3) and diacylglycerol (DG), which change the currents of K(and Ca^2^(and increase brain excitability. Moreover, increased glutamate activates AMPA/KA receptors into the cell. As a result, this phenomenon removes Mg^2^(, which inhibits NMDA receptors, leading to an increase in Ca^2^(permeability into the postsynaptic neurons, excitotoxicity, and neuronal cell death (Segal, 1988; Scorza et al., 2009).

Cerebrolysin is a small molecule peptide extracted from the porcine brain (Khalili et al., 2017) and has been previously used as a nootropic drug (Plosker and Gauthier, 2009). In another study, cerebrolysin was confirmed to reduce neuronal cell death and increase neurogenesis and brain functions in diverse brain diseases, such as mouse closed head injury (mCHI) and stroke (Zhang L. et al., 2013; Zhang Y. et al., 2013). In addition, it has been shown in many previous studies that cerebrolysin promotes neuroprotection and neurogenesis by increasing the expression of factors such as NGF and brain-derived neurotrophic factor (BDNF) (Rockenstein et al., 2015; Shishkova et al., 2015; Alvarez et al., 2016). In this way, cerebrolysin has shown positive effects in several brain diseases. However, the effects of cerebrolysin on epilepsy are still unknown.

BDNF, as a neurotrophin, promotes nerve differentiation, survival, and neurogenesis (Noble et al., 2011). BDNF simulates the growth and differentiation of new neurons (Alderson et al., 1990; Knusel and Hefti, 1991) and promotes neuronal survival (Hofer and Barde, 1988) and long-term potentiation (LTP) (Korte et al., 1996). BDNF is abundantly expressed throughout the central nervous system (CNS) (Lommatzsch et al., 1999).

In our lab, we hypothesized that BDNF might be increased by cerebrolysin, which would lead to reduced neuronal death. Therefore, this study investigated the effects of cerebrolysin in epilepsy, with BDNF signaling as our primary candidate for promoting neuroprotection after injury.

## Materials and Methods

### Ethics Statement

This study was exhaustively approved according to the rules of the Laboratory Animals Guide and Laboratory Animals published by the National Institutes of Health (NIH). Animal experiments were performed according to the criteria of the Committee on Animal Habitation (Protocol # Hallym 2018-73). We made every effort to minimize the pain of the animals, which were ultimately sacrificed by isoflurane anesthesia.

### Experimental Animals

This experiment used Sprague–Dawley male rats (250–350 g, DBL Co., Korea) aged 8 weeks. The animal rooms were kept at a constant humidity (55 ± 5%) and room temperature (22 ± 2°C). The room’s lighting was set to automatically switch on at 12 h intervals (on at 6:00 and off at 18:00). This guideline was designed based on the ARRIVE (Animal Research: Reporting *in Vivo* Experiments) guidelines.

### Seizure Induction

To confirm the effect of cerebrolysin on neuronal death after pilocarpine-induced seizure, the rats were administered lithium chloride (127 mg/kg, i.p, Sigma-Aldrich Co., St. Louis, MO, United States) 19 h before the administration of pilocarpine. Scopolamine (2 mg/kg, i.p., Sigma-Aldrich Co., St. Louis, MO, United States) was administered 30 min before the administration of pilocarpine (Biagini et al., 2009). Thirty minutes after scopolamine administration, status epilepticus (SE) was induced by the intraperitoneal administration of pilocarpine (25 mg/kg, i.p., Sigma-Aldrich Co., St. Louis, MO, United States). SE is observed according to the presence of five symptoms (1. mouth and facial movement, 2. head nodding, 3. forelimb clonus, 4. rearing with forelimb clonus, and 5. rearing and falling with forelimb clonus) that occur progressively in Racine’s method. The animals were placed in individual cages for ease of observation. SE usually occurred within 20–30 min after pilocarpine injection (Persinger et al., 1988). Diazepam (10 mg/kg, i.p., Valium, Hoffman la Roche, Neuilly sur-Seine, France) was injected intraperitoneally 2 h after the last Racine’s stage occurred (Biagini et al., 2001). If the animals presented consistent recurrent seizures, additional diazepam was injected (2 mg/kg, i.p.) (Kim et al., 2013).

### Cerebrolysin Administration

Experimental groups were classified into four groups: sham-vehicle, sham-cerebrolysin, seizure-vehicle, and seizure-cerebrolysin. To evaluate the effect of cerebrolysin on pilocarpine-induced seizures, cerebrolysin groups were injected with cerebrolysin (2.5 ml/kg, i.p., Ever Neuro Pharma, Unterach, Austria) intraperitoneally daily for 1 week, 2 h after a seizure induction, and vehicle groups were injected with 0.9% saline intraperitoneally in the same way. Also, to evaluate the anticonvulsant effect of cerebrolysin on pilocarpine-induced seizure, cerebrolysin groups were injected with cerebrolysin (2.5 ml/kg, i.p.) 10 min before pilocarpine injection. The present study used this cerebrolysin concentration since several works have demonstrated a significant neuroprotective effect after brain insult (Zhang Y. et al., 2013; Liu et al., 2017; Zhang et al., 2019).

### Brain Sample Preparation

Animals were sacrificed at 1 week after a seizure. Animals were injected with urethane (1.5 g/kg, i.p.) as anesthesia. After completely entering an anesthetic state, the animals were perfused with 0.9% saline and then 4% paraformaldehyde. The brains were harvested quickly and accurately and were fixed with 4% paraformaldehyde for 1 h. After fixation, the brains were immersed in a 30% sucrose solution as a cryoprotectant for 2 days (Vinet et al., 2016). Two days later, when the brains had sunk, the brains were frozen with a cryostat. The brains were then cut to a thickness of 30 μm on the cryostat, and the tissue was stored in a stock solution until histological evaluation was performed.

### Microscope Equipment

Microscopy images were obtained with an Olympus IX70 microscope (Olympus, Shinjuku-ku, Tokyo) equipped with a U-HGLGPS (Olympus, Shinjuku-ku, Tokyo) and an INFINITY3 digital camera (Olympus, Shinjuku-ku, Tokyo). We obtained the image using the INFINITY ANALYZE software.

### Detection of Live Neurons

Live neurons were evaluated by staining for neuronal nuclei (NeuN) to confirm the effect of cerebrolysin on pilocarpine-induced seizure. Following the brain cryostat section, we stained the cut tissue. After precleaning to eliminate the remaining blood cells in the tissues, we put the tissues in monoclonal mouse anti-NeuN antiserum (diluted 1:500, Billerica, Millipore Co., MA, United States) and kept them overnight for 16 h at 4°C. Sixteen hours later, the tissues were placed in anti-mouse IgG (diluted 1:250, Burlingame, Vector, CA, United States) for 2 h at room temperature and then placed in an ABC complex solution (Burlingame, Vector, CA, United States) for 2 h at room temperature. Then, the samples were transferred to slides after 3,3′-diaminobenzidine (DAB ager, Sigma-Aldrich Co., St. Louis, MO, United States) coloring for 1.5 min. Slides were then dried and mounted using Canada balsam. The tissues were observed through an Axioscope microscope. Live neurons were averaged by blind quantification. Live neurons were quantified in the stratum pyramidale (SP) of hippocampal cornu ammonis1 (CA1) and CA3 and expressed as the density (cell count/mm^2^).

### Detection of Microglial Cells

Microglial cells were evaluated by ionized calcium-binding adaptor molecule 1 (Iba1) to confirm the effect of cerebrolysin on pilocarpine-induced seizure. Following brain cryostat sectioning, we stained the cut tissue. After precleaning to eliminate the remaining blood cells in the tissues, we put the tissues in monoclonal goat anti-Iba1 antiserum (diluted 1:500, AbD Serotec, United Kingdom) and kept them overnight for 16 h at 4°C. Sixteen hours later, the tissues were placed in Alexa Fluor 594-conjugated donkey anti-goat IgG secondary antibody (diluted 1:250, Invitrogen, Grand Island, NY, United States) for 2 h at room temperature. Then, they were put on the slides. The slides were dried and mounted with DPX (Sigma-Aldrich Co., St. Louis, MO, United States), and the tissues were observed through an Axioscope microscope. Microglial cells were quantified in the stratum oriens (SO), stratum pyramidale (SP), and stratum radiatum (SR) of the hippocampal CA1 and CA3. Microglial cells were expressed as their density (cell count/mm^2^).

### Detection of Astroglial Cells

Astroglial cells were evaluated by the glial fibrillary acidic protein (GFAP) to confirm the effect of cerebrolysin on pilocarpine-induced seizure. Following brain cryostat sectioning, we stained the cut tissue. After precleaning to eliminate the remaining blood cells in the tissues, we put the tissues in monoclonal rabbit anti-GFAP antiserum (diluted 1:1,000, AbD Serotec, United Kingdom) and kept them overnight for 16 h at 4°C. Sixteen hours later, the tissues were placed in Alexa Fluor 488-conjugated donkey anti-rabbit IgG secondary antibody (diluted 1:250, Invitrogen, Grand Island, NY, United States) for 2 h at room temperature. Then, the samples were placed on the slides. The slides were dried and mounted with DPX (Sigma-Aldrich Co., St. Louis, MO, United States), and the tissues were observed through an Axioscope microscope. Astroglial cells were then quantified in the stratum oriens (SO), stratum pyramidale (SP), and stratum radiatum (SR) of hippocampal CA1 and CA3. Astroglial cells were expressed as their density (cell count/mm^2^).

### Detection of Apoptotic Cells

Apoptotic cells were evaluated by cleaved caspase-3 staining to confirm the effect of cerebrolysin on pilocarpine-induced seizure. Following brain cryostat sectioning, we stained the cut tissue. After precleaning to eliminate the remaining blood cells in the tissues, we put the tissues in polyclonal rabbit anticleaved caspase-3 antiserum (diluted 1:200, Cell signaling, Danvers, MA, United States) and kept them overnight for 16 h at 4°C. Sixteen hours later, the tissues were placed in Alexa Fluor 488 donkey anti-rabbit IgG secondary antibody (diluted 1:250, Invitrogen, Grand Island, NY, United States) for 2 h at room temperature. Then, the samples were placed on slides. The slides were dried and mounted with DPX (Sigma-Aldrich Co., St. Louis, MO, United States). The tissues were then observed through an Axioscope microscope. Apoptotic cells were counted by blind quantification in the hippocampal CA1 and CA3 regions.

### Western Blotting

To verify the protein level of BDNF in the vehicle and cerebrolysin groups, we performed a Western blotting analysis. The bilateral hippocampus was obtained and homogenized in a RIPA buffer consisting of 10 mM Tris-HCl (pH 7.4), 1% Non-idet P-40, 150 mM NaCl, 0.5% sodium deoxycholate, and 0.1% SDS. The homogenized hippocampus was centrifuged at 14,000 × *g* for 20 min at 4°C, and the supernatant was harvested. The harvested supernatant was incubated at 100°C for 10 min and stored at −80°C in an ultra-low freezer until use. The protein composition within the hippocampus was measured by a Bradford protein assay. Hippocampal proteins were diluted in an SDS electrophoresis sample buffer, separated on a 14% SDS-polyacrylamide gel, and then transferred to a PVDF (polyvinylidene difluoride) membrane. Non-specific binding was prevented by using 5% skim milk and 5% BSA (TNF-α) in TBST (50 mM Tris-HCl, pH 7.5, 0.1% Tween 20 and 150 mM NaCl) for over 1 h at room temperature. Protein-transferred membranes were incubated on primary antibodies (BDNF, ab108317, diluted 1:1,000, Abcam, TrkB, #4603, diluted 1:2,000, Cell signaling, phospho-TrkB (p-TrkB), ABN1381, diluted 1:1,000, Millipore, phospho-CREB (p-CREB), #9198, diluted 1:1,000, Cell signaling, TNF-α, ab6671, diluted 1:500, Abcam) overnight at 4°C in an incubator. After primary antibody incubation, the membranes were washed three times for 5 min in TBST. Afterward, the primary anti-BDNF, anti-TrkB, anti-phospho-TrkB, anti-phospho-CREB, and anti-TNF-α-reacted membranes were incubated for 1 h in anti-rabbit IgG secondary antibody conjugated with horseradish peroxidase (HRP, LF-SA8002, diluted 1:5,000, Ab Frontier). Last, to visualize the protein concentration, we used an ECL (enhanced chemiluminescence) solution (Cat.P90720, Millipore) before observation. The ECL solution-mounted membrane was made to react using a chemiluminescence imaging system device (Amersham imager 680 machine, GE healthcare). All data were analyzed by Image J.

### Statistical Analysis

We used non-parametric tests to determine the statistical significance between the experimental groups. The data of four groups were analyzed by a Kruskal–Wallis test and Bonferroni *post hoc* analysis, and the data from the two groups were analyzed by a Mann–Whitney U test. The data are expressed as the standard error of the mean (SEM) and were regarded as significant when the difference was *p* < 0.05.

## Results

### Experimental Procedure and Seizure Grade at Cerebrolysin Treatment

**FIGURE 1:**
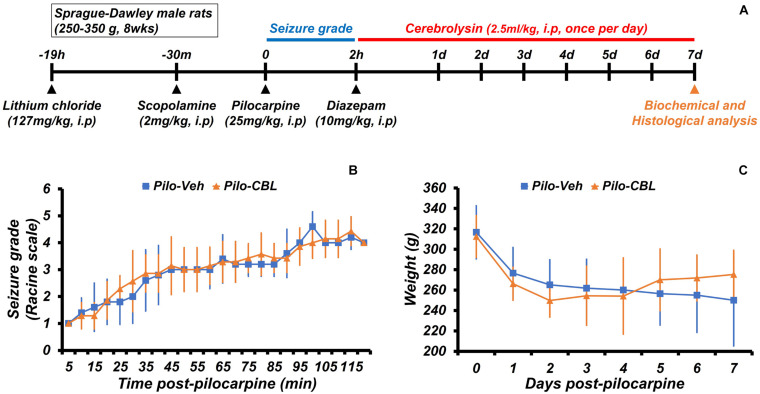
Experimental procedure and seizure grade during cerebrolysin treatment. The experimental paradigm of this study and the seizure grade according to the Racine stage. **(A)** The experimental paradigm of this study. After a seizure was induced for 2 h by pilocarpine, cerebrolysin was administered once a day at a concentration of 2.5 ml/kg for 1 week. **(B)** A graph confirming the average value of the seizure grade based on the Racine stage after pilocarpine administration. **(C)** A graph of the average body weight for 1 week after pilocarpine-induced seizure; *n* = 5–7 for each seizure group.

We confirmed the neuroprotective effects of cerebrolysin treatment on seizure-induced neuronal death. Cerebrolysin was injected for 1 week after pilocarpine-induced seizure (Figure 1A). Both groups were confirmed to have equally induced seizures (Figure 1B). Only seizure-induced rats were used in the experimental groups. In both groups, there was no difference in weight change during the 1-week period after a seizure induction (Figure 1C). In addition, to test whether cerebrolysin treatment has an anticonvulsant effect, we injected cerebrolysin 10 min before pilocarpine injection. There was no difference in seizure grade between the vehicle- and cerebrolysin-treated groups (Supplementary Figure S1).

### Cerebrolysin Increases the Density of Live Neurons After Pilocarpine-Induced Seizure

**FIGURE 2:**
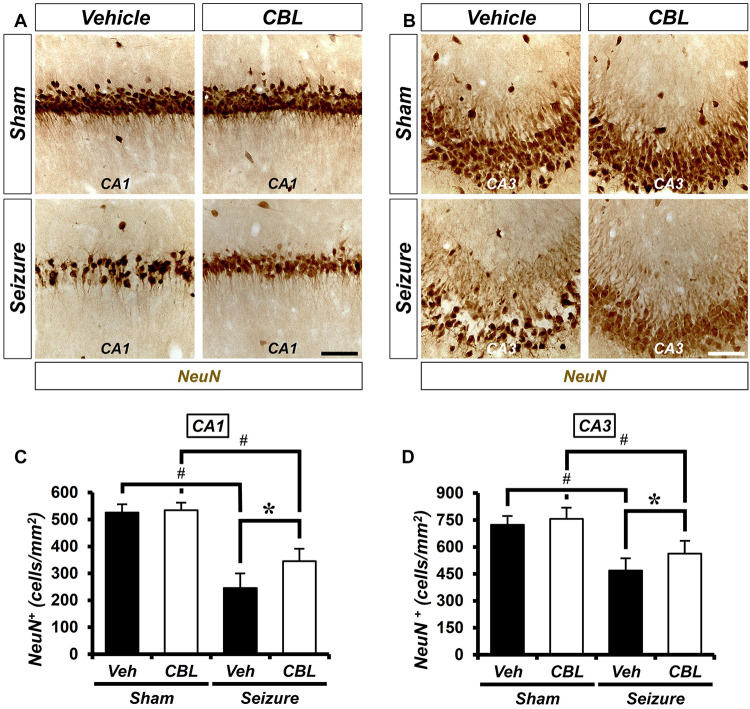
Cerebrolysin increases the density of live neurons after pilocarpine-induced seizure. The administration of cerebrolysin decreases neuronal death after pilocarpine-induced seizure. **(A,C)** NeuN (+) neurons in the hippocampal CA1 and CA3 regions. After a seizure, the administration of cerebrolysin for 1 week increased the density of live neurons in the hippocampal CA1 and CA3 regions compared with the seizure-vehicle groups. Scale bar = 100 μm. **(B,D)** Graphs that show the density of live neurons. The data are the mean ± SEM, *n* = 5 from each sham group. *N* = 5–7 for each seizure group. *p < 0.05 vs. vehicle-treated group; ^#^*p* < 0.05 vs. sham-operated group [Kruskal–Wallis test with *post hoc* test: **(C)** Chi square = 17.722, *df* = 3, *p* = 0.001, **(D)** Chi square = 17.024, *df* = 3, *p* = 0.001].

We conducted neuronal nuclei (NeuN) staining to confirm the effects of cerebrolysin on neuronal survival after a seizure. We sacrificed the animals at 1 week after inducing seizure and quantified the density of their live neurons in the hippocampus. As a result, after comparing the density of the live neurons of the seizure-vehicle and seizure-cerebrolysin groups, we found that the density of live neurons was increased in the group treated with cerebrolysin in the hippocampal CA1 and CA3 regions, rather than the seizure-vehicle group (Figure 2). The data are the mean ± SEM (*n* = 5) for each sham group, and *n* = 5–7 for each seizure group {^∗^*p* < 0.05 vs. vehicle-treated group; ^#^*P* < 0.05 vs. sham-operated group [Kruskal–Wallis test with *post hoc* test: (CA1) Chi square = 17.722, *df* = 3, *p* = 0.001 (CA3), Chi square = 17.024, *df* = 3, *p* = 0.001]}.

### Cerebrolysin Decreases the Density of Glial Cells After Pilocarpine-Induced Seizure

**FIGURE 3:**
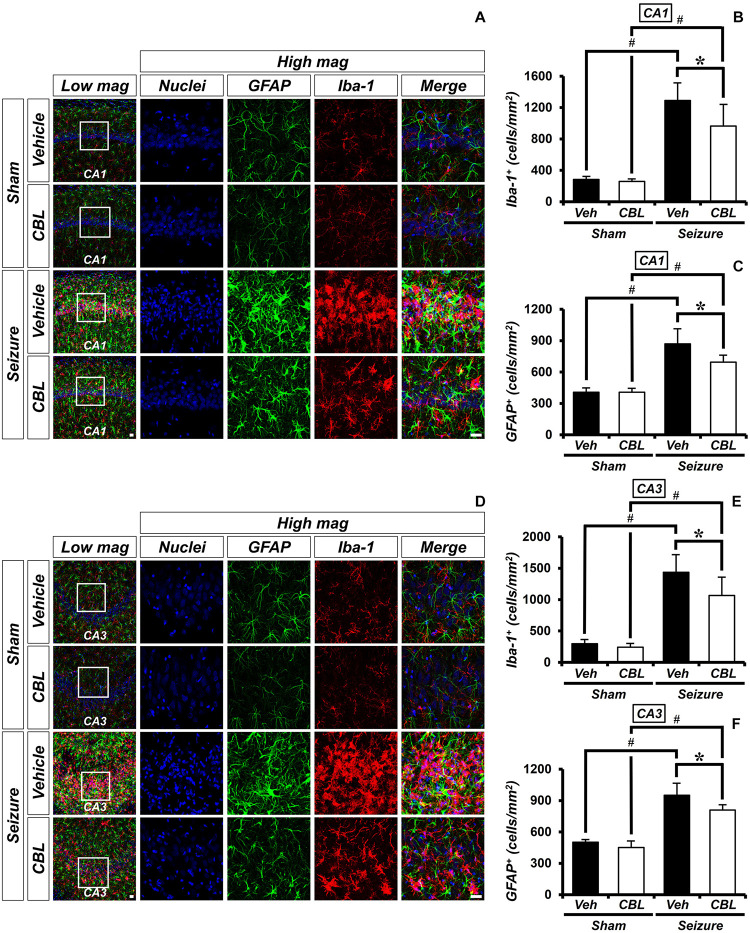
Cerebrolysin decreases the density of glial cells after pilocarpine-induced seizure. The administration of cerebrolysin decreased the density of glial cells after pilocarpine-induced seizure. **(A,D)** Iba-1 (red), GFAP (green), and DAPI (blue) in the hippocampal CA1 **(A)** and CA3 **(D)** regions. The administration of cerebrolysin after a seizure decreased the density of glial cells in the hippocampal CA1 and CA3 regions compared to the seizure-vehicle groups. Scale bar = 20 μm. **(B,C,E,F)** A graph of the density of glial cells according to the standard. The data are the mean ± SEM, *n* = 5, from each sham group; *n* = 5–7 for each seizure group. *p < 0.05 vs. vehicle-treated group; ^#^*p* < 0.05 vs. sham-operated group [Kruskal–Wallis test with *post hoc* test: **(B)** Chi square = 17.244, *df* = 3, *p* = 0.001, **(C)** Chi square = 17.153, *df* = 3, *p* < 0.001, **(E)** Chi square = 17.456, *df* = 3, *p* = 0.001, **(F)** Chi square = 18.292, *df* = 3, *p* < 0.001].

Glial activation is increased not only during seizure but also after other diseases (Hong et al., 2018; Lee S. H. et al., 2018; Kho et al., 2019). Inflammation was triggered by activated glial cells after a seizure. To determine the effect of cerebrolysin on the density of glial cells, we performed ionized calcium-binding adaptor molecule 1 (Iba-1) and GFAP staining, which are immunofluorescent stains used to confirm microglia and astroglial cells, respectively. There was little staining of glial cells in the sham-treated groups. On the other hand, we found that the group treated with cerebrolysin showed a decreased density of glial cells in the hippocampal CA1 and CA3 regions compared to the seizure-vehicle group (Figure 3). The data are the mean ± SEM, *n* = 5, for each sham group, and *n* = 5–7 for each seizure group {^∗^*p* < 0.05 vs. vehicle-treated group; ^#^*p* < 0.05 vs. sham-operated group [Kruskal–Wallis test with *post hoc* test: (Iba-1, CA1) Chi square = 17.244, *df* = 3, *p* = 0.001, (GFAP, CA1) Chi square = 17.153, *df* = 3, p0.001 (Iba-1, CA3) Chi square = 17.456, *df* = 3, *p* = 0.001, (GFAP, CA3) Chi square = 18.292, *df* = 3, *p* < 0.001]}.

### Cerebrolysin Decreases Levels of TNF-α After Pilocarpine-Induced Seizure

**FIGURE 4:**
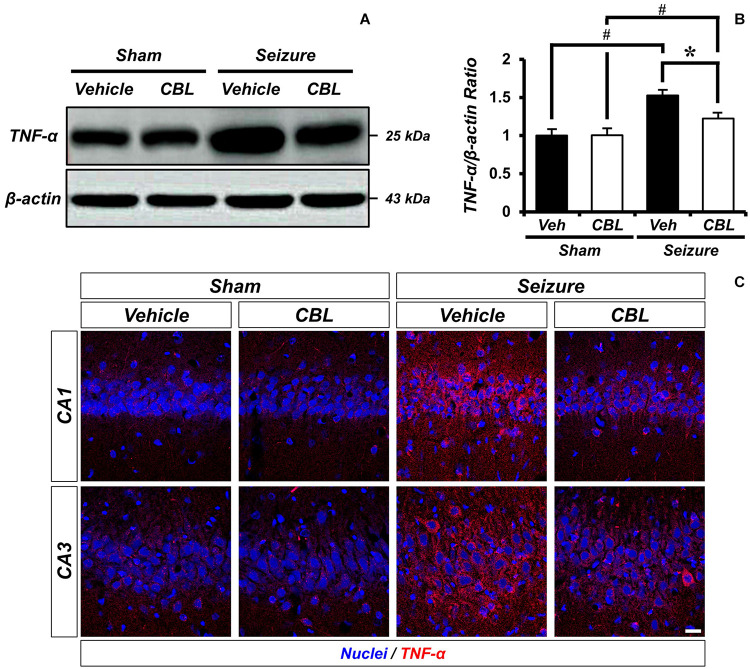
Cerebrolysin decreases level of TNF-α after pilocarpine-induced seizure. The administration of cerebrolysin decreased TNF-α after pilocarpine induced seizure. **(A)** indicates the level of TNF-α in the hippocampus. After seizure, the administration of cerebrolysin decreased TNF-α expression in the hippocampus compared to vehicle groups. **(B)** A graph of the TNF-α. **(C)** TNF-α (red) and DAPI (blue) in the hippocampal CA1 and CA3 regions. The data are mean ± SEM, *n* = 3–4 from each seizure group. *p < 0.05 vs. vehicle-treated group; ^#^*p* < 0.05 vs. sham-operated group (Kruskal–Wallis test with *post hoc* test: Chi square = 17.153, *df* = 3, *p* = 0.001).

Tumor necrosis factor-α (TNF-α) is a proinflammatory cytokine (Decourt et al., 2017). To confirm if suppressed glial activation via the administration of cerebrolysin reduces proinflammation, we confirmed the protein level of TNF-α by Western blot analysis after a seizure. In the present study, we found that the seizure group showed increased TNF-α expression in the hippocampus compared to the sham group. We also found that TNF-α expression was significantly reduced via the administration of cerebrolysin after a seizure (Figure 4). The data are the mean ± SEM, *n* = 3–4, for each seizure-experienced group [^∗^*p* < 0.05 vs. vehicle-treated group; ^#^*p* < 0.05 vs. sham-operated group (Kruskal–Wallis test with *post hoc* test: Chi square = 17.153, *df* = 3, *p* = 0.001)].

### Cerebrolysin Decreases the Number of Apoptotic Cells After Pilocarpine-Induced Seizure

**FIGURE 5:**
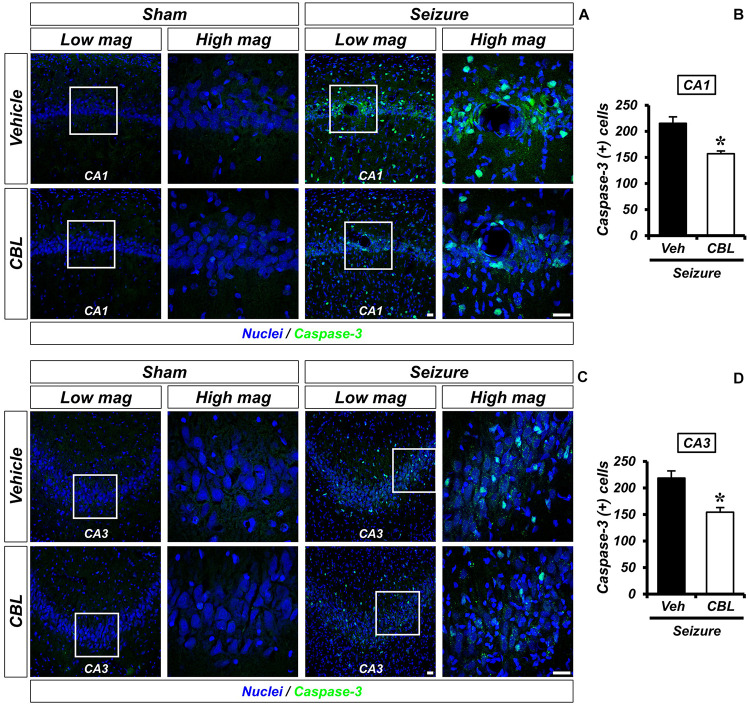
Cerebrolysin decreases the number of apoptotic cells after pilocarpine-induced seizure. The administration of cerebrolysin decreases apoptosis after pilocarpine-induced seizure. **(A,C)** Cleaved caspase-3 (green) in the hippocampal CA1 and CA3 regions. After a seizure, the administration of cerebrolysin for 1 week decreased the number of apoptotic cells in the hippocampal CA1 and CA3 regions compared to the seizure-vehicle groups. Scale bar = 20 μm. **(B,D)** A graph that shows the number of apoptotic cells. The data are the mean ± SEM, *n* = 5–7 for each seizure group. *p < 0.05 [Mann–Whitney *U*-test: **(B)** *z* = 2.842, *p* = 0.03, **(D)** *z* = 2.517, *p* = 0.01].

Cleaved caspase-3 staining is an immunofluorescent staining method used to confirm apoptosis. We performed cleaved caspase-3 staining to verify the effect of cerebrolysin on apoptosis after a seizure. There was no caspase-3 activation observed in the sham-operated groups. The seizure-experienced group showed an increased level of caspase-3 activation in the hippocampal CA1 and CA3 regions. However, the administration of cerebrolysin showed a decreased number of caspase-3-positive cells in the hippocampal CA1 and CA3 regions compared to the seizure-vehicle group (Figure 5). The data are the mean ± SEM, *n* = 5–7, for each seizure group {^∗^*p* < 0.05 [Mann–Whitney U test: (CA1) *z* = 2.842, *p* = 0.03, (CA3) *z* = 2.517, *p* = 0.01]}.

### Cerebrolysin Increases Levels of BDNF After Pilocarpine-Induced Seizure

**FIGURE 6:**
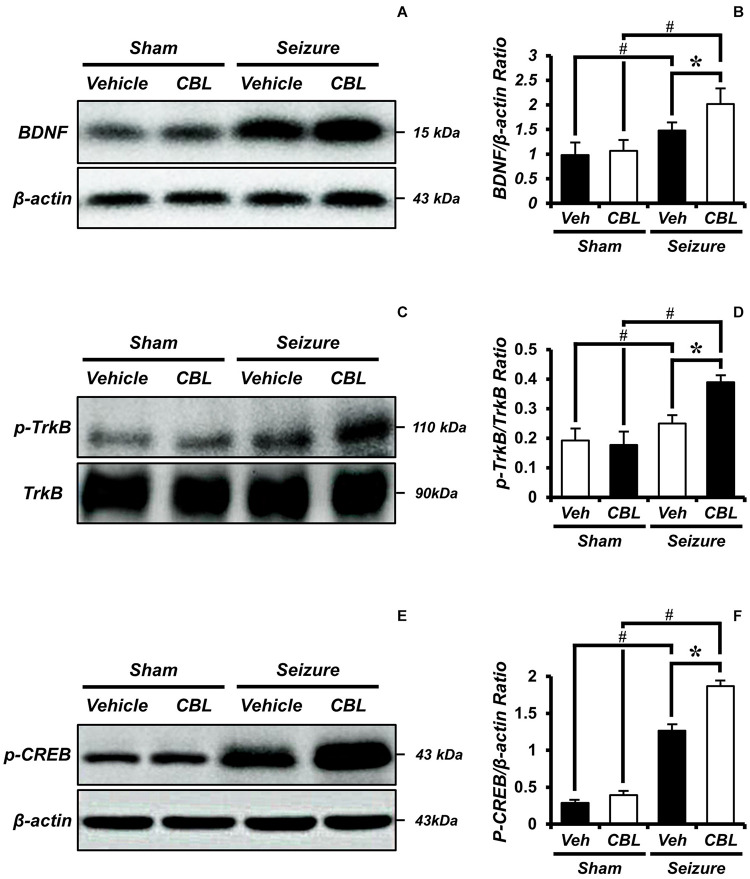
Cerebrolysin increases the levels of brain-derived neurotrophic factor (BDNF), phospho-tyrosine kinase receptor B (p-TrkB), and phospho-cAMP-response-element-binding (p-CREB) after pilocarpine-induced seizure. The administration of cerebrolysin increased BDNF after pilocarpine-induced seizure. **(A)** The level of BDNF in the hippocampus. After a seizure, the administration of cerebrolysin increased BDNF expression in the hippocampus compared to the seizure-vehicle groups. **(B)** A graph of the BDNF. **(C)** The level of p-TrkB in the hippocampus. After a seizure, the administration of cerebrolysin increased p-TrkB expression in the hippocampus compared to the seizure-vehicle groups. **(D)** A graph of the p-TrkB. **(E)** The level of p-CREB in the hippocampus. After a seizure, the administration of cerebrolysin increased p-CREB expression in the hippocampus compared to the vehicle groups. **(F)** A graph of the p-CREB. The data are the mean ± SEM, *n* = 3–4, for each seizure group. *p < 0.05 vs. vehicle-treated group; ^#^*p* < 0.05 vs. sham-operated group [Kruskal–Wallis test with *post hoc* test: **(B)** Chi square = 9.705, *df* = 3, *p* = 0.021, **(D)** Chi square = 9.029, *df* = 3, *p* = 0.029, **(F)** Chi square = 11.895, *df* = 3, *p* = 0.008].

BDNF is regarded as a potent neural modulator, which is beneficial to neuronal functions and promotes neuroprotection (Chen et al., 2013). To confirm the increase in BDNF and determine whether the increase was mediated by cerebrolysin, we performed a Western blot analysis to confirm the level of the brain-derived neurotropic factor (BDNF) after a seizure. By comparing the BDNF expressions of the sham and seizure groups, we confirmed that the seizure group experienced increased BDNF expression in the hippocampus compared to the sham group. Moreover, comparing the BDNF expression of the seizure-vehicle and seizure-cerebrolysin groups, we demonstrated that the administration of cerebrolysin increased BDNF expression in the hippocampus is greater than in the seizure-vehicle group (Figures 6A,B). The data are the mean ± SEM, *n* = 3–4, for each seizure group [^∗^*p* < 0.05 vs. vehicle-treated group; ^#^*p* < 0.05 vs. sham-operated group. (Kruskal–Wallis test with *post hoc* test: Chi square = 9.705, *df* = 3, *p* = 0.021)].

#### Cerebrolysin Increases Levels of p-TrkB/Tyrosine Kinase Receptor B After Pilocarpine-Induced Seizure

Tyrosine kinase receptor B (TrkB) is known as a receptor for BDNF (Budni et al., 2015). To evaluate BDNF receptor activation, we analyzed the levels of p-TrkB/TrkB by Western blot after a seizure. In the present study, we found that the seizure-experienced group showed increased levels of p-TrkB/TrkB expression in the hippocampus compared to the sham group. We found that cerebrolysin further increased the level of p-TrkB/TrkB expression in the hippocampus compared to the vehicle-treated group after seizure (Figures 6C,D). The data are the mean ± SEM, *n* = 3–4 for each seizure group [^∗^*p* < 0.05 vs. vehicle-treated group; ^#^*p* < 0.05 vs. sham-operated group (Kruskal–Wallis test with *post hoc test*: Chi square = 9.029, *df* = 3, *p* = 0.029)].

#### Cerebrolysin Increases Levels of p-CREB After Pilocarpine-Induced Seizure

Phospho-cAMP-response-element-binding (p-CREB) is a transcription factor present downstream of BDNF that promotes neuronal protection and cell survival (Walton and Dragunow, 2000; Li et al., 2018). We evaluated the levels of p-CREB by Western blot analysis after seizure. We found that the seizure-experienced group showed increased levels of p-CREB expression in the hippocampus compared to the sham-operated group. Moreover, as seen in the p-TrkB/TrkB expression, the administration of cerebrolysin further increased the p-CREB expression in the seizure-experienced group (Figures 6E,F). The data are the mean ± SEM, *n* = 3–4 for each seizure group [^∗^*p* < 0.05 vs. vehicle-treated group; ^#^*p* < 0.05 vs. sham-operated group (Kruskal–Wallis test with *post hoc* test: Chi square = 11.895, *df* = 3, *p* = 0.008)].

## Discussion

In the present study, we verified that cerebrolysin exerts powerful neuroprotective effects after pilocarpine-induced seizure. Seizure is one of the most common neurological diseases, but methods for preventing the cell death mechanisms that occur post-seizure and repairing this injury after a seizure remain uncertain. Seizures cause serious damage to the hippocampus, and neuronal death is ultimately caused by a series of cell death cascades involving excessive inflammation, glial activation, apoptosis, oxidative stress, and zinc accumulation (Kim et al., 2012; Jeong et al., 2017). Here, we focused on the rescue of seizure-induced neurological damage with cerebrolysin.

Cerebrolysin is neuropeptide extracted from porcine brains and has been used as a nootropic drug (Plosker and Gauthier, 2009). It is known that cerebrolysin can pass intact across the blood–brain barrier (BBB) (Muresanu et al., 2015; Bornstein et al., 2018). When cerebrolysin is administered, it is not known how much cerebrolysin reaches the brain. However, with status epilepticus, there is a known loss of integrity at the BBB with the entry of proteins such as albumin (Kim et al., 2015; Lee et al., 2017; Mendes et al., 2019). Disruption of the BBB, therefore, enables the entry of cerebrolysin into the brain. Furthermore, several studies have shown that the neuropeptide cerebrolysin can pass intact across the BBB (Muresanu et al., 2015; Bornstein et al., 2018). Cerebrolysin demonstrated significant neuroprotective effects and increased neurogenesis after brain insults when injected intraperitoneally (Zhang L. et al., 2013; Liu et al., 2017). Several previous studies have demonstrated that under head trauma and ischemic conditions, the administration of cerebrolysin attenuates brain damage (Zhang L. et al., 2013; Zhang Y. et al., 2013). In addition, previous studies have shown that cerebrolysin increases the level of the BDNF by inhibiting the activity of glycogen synthase kinase-3 beta (GSK-3β) (Alvarez et al., 2016). It has also been shown that enhancing BDNF expression decreases neuronal damage and inflammation and increases neurogenesis (Wada et al., 2003; Chen et al., 2013; Rockenstein et al., 2015). However, the effects of cerebrolysin on seizure-induced neuronal death are not well known. Based on these previous findings, we hypothesized that the administration of cerebrolysin after pilocarpine-induced seizure would attenuate neuronal death by increasing levels of BDNF.

To evaluate the neuroprotective effects of cerebrolysin, we first injected cerebrolysin once a day for 1 week after a seizure and then confirmed the neuroprotective effects. Then, we performed several histological evaluations after pilocarpine-induced seizure. We next performed staining for NeuN, a specific marker of live neurons, and cleaved caspase-3, a specific marker for apoptotic cells, to confirm the effects of cerebrolysin on neuronal death after pilocarpine-induced seizure. It is already well known that the number of live neurons decreases after a seizure (Kim et al., 2012; Jeong et al., 2017; Lee S. H. et al., 2018). Also, it is already well established that the number of apoptotic cells increases after a seizure (Lee J. M. et al., 2018; Li et al., 2019). To confirm the effects of cerebrolysin on neuronal death in a damaged hippocampus following a seizure, the density of live neurons was recorded to determine whether cerebrolysin treatment could rescue neuronal damage after a seizure. In the results, the sham-operated groups showed no differences in the density of their NeuN positive neurons between the vehicle and cerebrolysin groups. However, in the seizure-operated groups, the cerebrolysin-administered group showed a significant increase in the density of NeuN-positive neurons in hippocampal CA1 and CA3 compared to the vehicle group.

In addition to directly damaging neurons, other cells in the region are also affected by seizure. Epileptic seizure-induced neuroinflammation is triggered by activated glial cells, which include microglia and astroglia (Scorza et al., 2009). The damage caused by seizures is a trigger for glial activation (Scorza et al., 2009). If excessive inflammation persists, it promotes subsequent deleterious effects, such as cellular damage and neurotoxicity (Peng et al., 2015; Stein et al., 2017). We proceeded to stain for Iba-1, a specific marker for microglia, and GFAP, a specific marker for astroglia, to assess the effect of cerebrolysin on the density of glial cells after a seizure. We confirmed the presence of glial cells in the hippocampus after a seizure. In the sham-operated groups, there was no observable difference in the density of microglial cells between the vehicle and cerebrolysin groups. However, in the seizure-operated groups, the cerebrolysin-administered group showed a significant decrease in the density of microglial cells in the hippocampal CA1 and CA3 regions compared to the vehicle group. The density of astroglial cells in the sham-operated groups showed no difference between the vehicle and cerebrolysin groups. However, in the seizure-operated groups, the cerebrolysin-administered group showed a significantly attenuated density of astroglial cells in the hippocampal CA1 and CA3 regions compared to the vehicle group.

Pilocarpine-induced seizure triggers glial activation and promotes the production of various inflammatory mediators, thus initiating a cascade of inflammatory processes in the hippocampus (Shapiro et al., 2008; Vezzani et al., 2011). The release of pro-inflammatory molecules can aggravate neuronal excitability and disturb the normal physiological functions of the glia, which perturbate glial–neuronal communications. In the pilocarpine-induced seizure model, the density of microglial cells thus contributes to decreasing the seizure threshold and compromising neuronal survival (Vezzani et al., 2008; Riazi et al., 2010). In the present study, we found that cerebrolysin administration decreased the density of microglial cells after a seizure. In addition, we evaluated the protein levels of TNF-α, a proinflammatory cytokine, by Western blot analysis to determine whether the cerebrolysin-induced reduction of microglial activation is associated with pro-inflammatory cytokine. Here, we found that the protein level of TNF-α in the cerebrolysin-administered group was decreased compared to that in the seizure-vehicle group.

Next, we confirmed that the observed reduction in neuronal death was associated with an increase in apoptosis induction through cleaved caspase-3 staining, which detects apoptotic cells. In the seizure-operated groups, the cerebrolysin-administered group showed a significant decrease in the number of apoptotic cells in the hippocampal CA1 and CA3 regions compared to the vehicle group. Cerebrolysin attenuates hippocampal neuronal death and apoptotic cell damage by increasing the concentration of the protease “furin,” which upregulates BDNF levels (Rockenstein et al., 2015). Increased BDNF elevates TrkB activity, which is associated with antiapoptosis signaling and inhibits neuronal apoptosis (Chen et al., 2013). Following the above logic, we assumed that this process caused the cerebrolysin group to decrease neuronal apoptosis and increase the density of live neurons compared to the vehicle group (Rockenstein et al., 2005).

**FIGURE 7:**
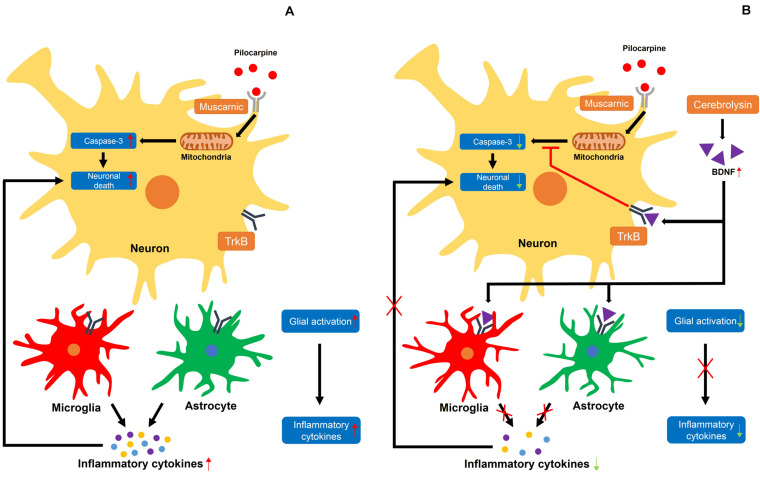
Proposed mechanism for the effects of cerebrolysin on seizure-induced neuronal death. **(A)** A schematic drawing that proposes the possible cellular pathways through which a seizure may induce neuronal death. **(A)** The damage caused by excessive neuronal excitation when pilocarpine is bound by a muscarinic receptor increases caspase-3-dependent neuronal death. In addition, activated glial cells secrete inflammatory cytokines, increasing neuronal death. **(B)** Increased BDNF levels by cerebrolysin can result in the inhibition of caspase-3 and glial activation, which are thought to occur following pilocarpine-induced seizure. A red X indicates inhibition.

Several studies have demonstrated that neurotrophic factors, including NGF and BDNF, are elevated after status epilepticus, which promotes the survival of neurons post-seizure (VonDran et al., 2014; Lima et al., 2015; Sanna et al., 2017). Thus, we evaluated the protein levels of BDNF by using a Western blot analysis to test whether an increase in neuroprotection after cerebrolysin administration is correlated with increases in BDNF. Here, we found that BDNF expression was increased in the seizure group compared with the sham operated group, as also shown by another group. Previous studies have already shown that BDNF is increased after pilocarpine-induced seizure (da Penha Berzaghi et al., 1993; Metsis et al., 1993; Schmidt-Kastner et al., 1996). This is considered to be a defense mechanism in the brain to protect against neuronal degeneration. However, this mechanism is insufficient for neuroprotection or the rescue of damaged neurons (Schmidt-Kastner et al., 1996). The present study found that cerebrolysin administration after pilocarpine-induced seizure further increased BDNF concentrations compared to the vehicle-treatment. In addition, cerebrolysin-administration increased the phosphorylation of TrkB, as well as the protein level of p-CREB downstream of the TrkB signal. This result supports our hypothesis that the increased BDNF concentrations by cerebrolysin contribute to neuroprotection in the hippocampus after status epilepticus (Figure 7).

It is well-established that neuronal death occurs in the brain after a seizure (Kim et al., 2012; Jeong et al., 2017; Lee S. H. et al., 2018), but the mechanisms through which this injury occurs remain elusive. When neuronal death occurs, an inflammatory response is initiated to restore tissue homeostasis and remove dead cells (Chapkin et al., 2009; Karin and Clevers, 2016). However, if the inflammatory response that occurs is excessive due to widespread neuronal death, the tissues are not repaired but can actually sustain further damage (Fontana, 2009; Park et al., 2011). Here, we identified an excessive inflammatory response after a seizure. The inflammatory cytokines released by an excessive inflammatory response increase glial activation (Watkins et al., 2014; Wu and Watabe, 2017). It was previously shown that BDNF has anti-inflammatory effects that counteract various inflammatory cytokines (Chen et al., 2013; Papathanassoglou et al., 2015; Liang et al., 2019). In this study, we used cerebrolysin, which increases BDNF, to reduce excessive inflammatory responses and confirmed that cerebrolysin administration after a seizure reduces inflammation and promotes neuronal survival.

Despite BDNF showing potential neuroprotective effects in stroke, traumatic brain injury, and Alzheimer’s disease, the therapeutic delivery of BDNF has many obstacles related to its short *in vivo* half-life and uncertain BBB permeability. BDNF is relatively unstable, and only a small fraction can cross the BBB after administration. If the level of administered BDNF is too small due to its short half-life and limited permeability, it may not show observable neurotrophic effects (Geral et al., 2013; Tanila, 2017; Wurzelmann et al., 2017). The commercial product, cerebrolysin^®^ (Ever Neuro Pharma, Unterach, Austria), is a mixture involving fragments of different neurotrophic factors, including BDNF. It has been demonstrated that BBB permeability promotes long-lasting BDNF supply to the brain (Sharma et al., 2012; Geral et al., 2013). Therefore, in the present study, we injected the neuropeptide cerebrolysin to increase BDNF levels in the brain.

## Conclusion

The present study found that the administration of cerebrolysin decreased seizure-induced neuronal death and glial activation by increasing BDNF levels. Although the precise mechanism through which cerebrolysin promotes increased BDNF production and downregulates microglial activation after a seizure remains unclear, the present study suggests that the administration of cerebrolysin can be a useful therapeutic agent to prevent neuronal death in this setting. However, substantial further research is needed to determine the mechanism by which cerebrolysin increases BDNF and promotes other neuroprotective outcomes.

## Data Availability Statement

All datasets presented in this study are included in the article/Supplementary Material.

## Ethics Statement

The animal study was reviewed and approved by the Committee on Animal Habitation.

## Author Contributions

DK researched the data and reviewed and edited the manuscript. BC reviewed and edited the manuscript. SL, AK, JJ, DH, BK, MP, HS, and HC researched the data. M-SL and SS contributed to the discussion and wrote, reviewed, and edited the manuscript. HS, HC, M-SL, and SS take full responsibility for the manuscript and its originality. All authors read and approved the final manuscript.

## Conflict of Interest

The authors declare that the research was conducted in the absence of any commercial or financial relationships that could be construed as a potential conflict of interest.

## Supplementary Material

Supplementary Figure 1 Pre-treatment of cerebrolysin has no anti-conversant effect on pilocarpine-induced seizure.
