# Supplementary material for: Effects of Cerebrolysin on Hippocampal Neuronal Death After Pilocarpine-Induced Seizure
Source: Front Neurosci. 2020 Oct 16;14:568813. doi: 10.3389/fnins.2020.568813 (PMC7596733; doi:10.3389/fnins.2020.568813)
Supplement: Supplementary Figure 1 — Pre-treatment of cerebrolysin has no anti-conversant effect on pilocarpine-induced seizure. [file Table_1.pdf]

Supplementary Figure 1

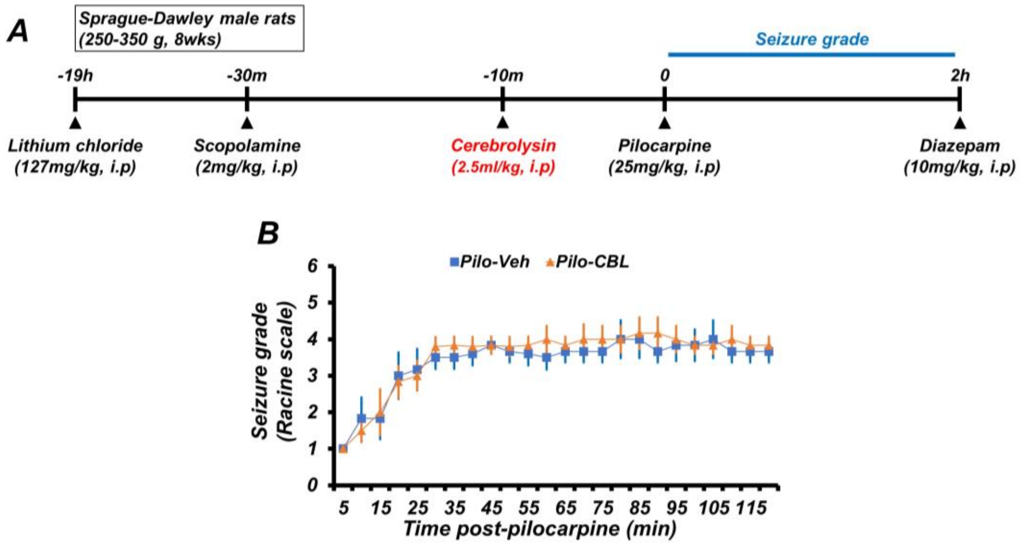

Figure S1. The experimental paradigm used in this study and the seizure grade according to the racine stage. Figure (A) indicates experimental paradigm of pre-treatment cerebrolysin. Cerebrolysin (2.5ml / kg) was administered 10 mins before pilocarpine injection following which, seizure is induced for 2 hours by pilocarpine. Figure (B) is a graph confirming the average score of the seizure grade based on the racine stage after pilocarpine administration. n=6 from each seizure group.
